# *In vitro* evaluation of some high yield potato (*Solanum tuberosum* L.) cultivars under imposition of salinity at the cellular and organ levels

**DOI:** 10.1016/j.sjbs.2021.12.040

**Published:** 2021-12-23

**Authors:** Isam M. Abu Zeid, Hemaid I.A. Soliman, Ehab M.R. Metwali

**Affiliations:** a Department of Biological Sciences, Faculty of Science, King Abdulaziz University, P.O. Box 139109, Jeddah 21323, Saudi Arabia; b Plant Genetic Resources Department, Desert Research Center, El-Matariya, Cairo 11753, Egypt; c Genetic Branch, Botany Department, Faculty of Agriculture, Suez Canal University, 41522 Ismailia, Egypt

**Keywords:** **P**otato (*Solanum tuberosum*L.), Callus, Regeneration, Microtubers, NaCl stress, Proline

## Abstract

Salinity and drought stress, which combines a lack of water and sodium toxicity, are more of the problems faced by plants and agricultural crops in newly reclaimed lands. Therefore, the direction of our research is to produce salinity-tolerant plants to increase the productivity of crops under conditions of salt stress. Potato callus was studied using different concentrations of NaCl (0.0, 50, 75, 100, 125, 150 and 200 mM). Shoot induction was obtained from callus treated with MS medium containing 4.0 and 5.0 mg l^−1^*TDZ + 0.5* mg l^−1^ GA3 with NaCl up to 125 mM and 150 mM for Rosetta and Victoria, respectively. When plantlets were cultured on MS medium containing 3.0 mg l^−1^ kinetin and 1.0 mg l^-1^paclobutrazol (PBZ) with 80 or 90 g l^−1^ sucrose after two months gave a good microtuber per explant of Rosetta and Victoria cultivar which gave number of microtuber/plantlet (1.85) and (2.40) when plantlets treated with 125 mM and 150 mM NaCl of Rosetta and Victoria cultivar, respectively. In general, the results were shown in each treatment of NaCl and that amounts of proline at 125 and 150 mMNaCl were significantly more than 0.0, 50, 75 and 100 mM NaCl. This result is related to the role of proline in the osmotic adjustment of a higher concentration of salinity. The results showed that the amounts of sodium increased with increasing the salt concentration, but the amount of potassium decreased and also increased the Na+/K+ ratio with increasing the salt concentration. This research is important for *in vitro* potato plant regeneration, which requires optimization before genetic transformation can be achieved.

## 1 Introduction

Potato (*Solanum tuberosum* L.) belongs to the family Solanaceae are grown worldwide. Potato is used as most important food for the largest number of worldwide population (Gowayed et al., 2017, Hossain, 1994) and is most suiting as biofortified crop to combat malnutrition in small and marginal farming community (Mitra, 2012). Potato tubers are a valuable food source since they are cheap and contain a variety of minerals (Martínez-Ballesta et al., 2010). The potato tuber dietary protein helps to reduce blood cholesterol by increasing the circulation of cholesterol levels (Gambuti et al., 2016). Hunger levels are on the rise, with nearly 690 million people suffering from undernourishment in 2019, or about 60 million more people than in 2014 (FAO, 2020), therefore, the interest in improving potato productivity in quantity and quality and raising its nutritional value has become a duty by agricultural organizations around the world in order to reduce the percentage of hunger.

In Saudi Arabia**,** the production area is approximately 19.149 thousand hectares, with a total production of 482.205 thousand tons (FAO, 2018). Environmental conditions (salt, drought, heat) in arid regions, such as Saudi Arabia, are generally adversely affect plant productivity including potato (Liao et al., 2016). Potato plant varieties a are usually very sensitive to environmental stresses, it has been classified as a moderately salt sensitive, thresholds ranging from 1.5 to 3.0 dS/m, due to short and shallow root systems. However, there exists a genotypic variation, among potato genotypes, to salt stress. There is a great loss in crop and plant product development when the potatoes grown in soil containing concentrations of 20–35 mm of sodium chloride (Rahman et al., 2008, Sajid and Aftab, 2014). Plant breeding techniques were heavily used during the green revolution to develop plants with desirable traits such as increased yield, higher nutritional value, and tolerance to abiotic and biotic stress (Ahmer et al., 2020). Screening and selection of potato cultivars growing in farm of diverse salt levels is an important step in breeding program to afford resources for enhancing the production of potato under harsh environments (Mousavi et al., 2020), however, examining the field performance of potato genotypes under these stresses are often inconclusive. Field trial is normally associated with non-uniform moisture availability and temperature fluctuations during the growing season. This method also requires a considerable amount of space, time, effort, equipment, and planting material (Metwali et al., 2015). Thus, plant breeders aim to develop plant salt tolerance by lowering the negative effect of salt stress on plant through different new biotech strategies such as tissue culture.

Previous studies indicated the effectiveness of tissue culture technology in the selection of cells tolerant to salt and also the interpretation of many cellular mechanisms that contribute to salt tolerance based on biochemical and physiological studies (Davenport et al., 2003, Gossett et al., 1996, Gu et al., 2004, Lutts et al., 2004, Olmos and Hellín, 1996, Wikandari et al., 2021). Culturing small plant growing tips, such as shoot apex, in the lab on specific nutrient media has a lot of potential for manipulating plants *in vitro* for the production of new varieties, and it is a great tool for studying many different aspects of plant development and growth under controlled conditions (Twaij et al. 2020). Shankhdhar et al., 2000, Miki et al., 2001 indicated the importance of these selected strains in producing regenerate salt tolerant plants. In potatoes in particular, the production of microtubers as alternative end product of micropropagation through *in vitro* tissue culture conditions is one of the most important goals of those interested in this field to produce and storage of valuable seed potato, germplasm storage and exchange (Badoni and Chauhan, 2009, Estrada et al., 1986). The microtubers are of length 10–12 mm and diameter ranging between 4 and 7 mm and weight not exceeding 280 mg (Ranalli, 2007).Since the production of the minitubers in greenhouse or pre-elite tubers in the field is considered and still a controversial, the alternative to this was to improve the production of the microtubers through tissue culture techniques (Coleman et al., 2001, Ranalli et al., 1994). Furthermore, it will be one of the limiting factors for potato improvement using other biotechnology techniques such as regeneration of potato transgenic plants and cryopreservation, without achieving an efficient propagation and homogeneity protocol *in vitro* for all varieties of potatoes (Idowu et al., 2009, Metwali et al., 2020, Reetika et al., 2019). Thus, the development of an effective protocol for micropropagated potato plantlets under normal and salt stress conditions will be one the main objectives of this research.

On the other hand, most research confirmed that proline accumulation is one of the most common metabolic responses to plants under salinity stress and are effective markers for selection)Sobieh et al., 2019). The hypothesis that proline acts as an alternate resource for carbon and nitrogen, helps to reduce oxidative damages, and stabilize DNA and membrane protein (Szabados and Savoure, 2010). Moreover, several nutrients such as K^+^ is an essential element that is important for the regulation of cell division, cell cycle progression (Wu et al., 2018) and pH homeostasis (Ahmad and Maathuis, 2014). Loss of K^+^ and Na^+^ toxicity that causes deficiency uptake of K^+^ from soil was reported in previous studies as a common phenomenon in plants under salt stress (Hanin et al., 2016, Hmidi et al., 2018). It is noticed that K^+^ and Na^+^ are key determinants of salinity tolerance due to their ability to determine tissue and cytosolic Na^+^/K^+^ ratios (Isayenkov and Maathuis, 2019)).

The research objective of this study was interested in finding an optimization protocol for *in vitro* multiplication and microtuberization of potato and mechanical way to tolerate salinity using morphological, physiological and biochemical markers at the cellular and organ levels. The developed protocol will assist in obtaining genetically improved potato cultivars.

## 2 Materials and methods

### 2.1 Plant material and surface sterilization

The tubers were cultured in the green house at 25 °C of potato (*Solunumtuberosum*) cvs. Victoria and Rosetta. Rosetta and Victoria cultivars considered as a sensitive cultivars to salinity. The nodal stem segments (2.0 cm length) were cut from field grown plants within 8–10 weeks. The surface of the node segment explants were first sterilized by washing under running tap water and the surface sterilized by immersion in 70% alcohol for one minute. After that, the explants were washed with sterile distilled water three times to remove traces of alcohol, then dipped in a 25% (volume / volume) sodium hypochlorite solution with two drops of liquid soap for 20 min and then finally rinsed five times with sterile distilled water. Explants were transferred to Murashige&Skoog's mineral salts containing 100 mg l^-1^myo-inositol, 10 mg l^−1^, thiamine-HC1, 0.5 mg l^-1^nicotinic acid, 0.5 mg l^-1^pyridoxine-HC1, 2.0 mg l^-1^glycine, 0.1 mg l^−1^ 6-benzylaminopurine (BAP), 0.05 mg l^−1^ indole-3-butyric acid (IBA), 1.0 mg l^-1^paclobutrazol (PBZ), 30 g l^-1^sucrose and 2.5 g l^-1^phytagel (Murashige and Skoog, 1962).

### 2.2 Callus induction

For the callus production, the leaves were removed from 3 to 4-weeks-*in vitro*-grown plantlets of potato (*Solanum tuberosum*L.), then cultured on Murashig and Skoog medium containing different concentrations of NAA, 2, 4-D in combination of kinetin and BAP. The MS medium was adjusted for pH 5.8. All samples are incubated in a culture room and kept in the dark at 25 ± 2° C. Callus appears 4 to 5 weeks after incubation of leaf explants. Callus is transferred every two weeks on the same medium. Callus formation (%) and relative water content (%) [(callus fresh weight- callus dry weight)/ (callus dry weight) × 100] were estimated after one month according to Farshadfar et al. (2012). The fresh weight of callus was dried in an oven at 65° C for 48 h to calculate the dry weight of the callus.

#### 2.2.1 *In vitro regeneration of potato* (*Solanum tuberosum* L.)

The calli were transferred to a new callus stimulation medium every 28 days and then incubated in the dark for further proliferation and multiplication for three months, the frequency of callus induction was determined and well-developed calli were selected and transplanted onto the regeneration media. MS medium contains BAP at a concentration of (0.5–5.0 mg l^−1^) alone or with a concentration of 0.5 mg l^−1^ kinetin and (1.0–5.0 mg l^−1^) TDZ alone or with a concentration of 0.5 mg l^−1^ GA3 for shoot induction when calli were incubated at 25 ± 2 °C with a light period of 16 hrs (Nistor et al., 2010).

#### 2.2.2 *In vitro* tuberization

After shoot proliferation, plantlets were grown in 250 ml jars containing 50 ml of ½ strength MS liquid medium containing different concentrations of (0.0–5.0 mg l^−1^) kinetin in combination with (1.0 mg l^−1^) paclobutrazol (PBZ) and different concentrations (50–90 g l^−1^) sucrose, the plantlets were incubated under complete darkness at 20 °C for 15 days and then transferred to at 25 ± 2 °C under complete darkness for the duration of 6 weeks. The percentage of microtubers number/ plantlets and microtubers weight/ plantlets (gm) was calculated according to Ali et al. (2018). The harvested microtubers were cold stored and used as minitubers seed for greenhouse.

### 2.3 Imposition of salinity

The MS medium which represent the best recommended combination derived from the establishment stage, as well as levels of salinity stress NaCl as (0.0, 50, 75, 100, 125, 150 and 200 mM) under laboratory conditions, using *in vitro* regeneration for both potato cultivars. Two pieces of callus were transferred into each concentration, each subculture were incubated for one month. The calli used in the experiment is gradually shifted to the different concentrations of NaCl. Each experiment was consisted of three replications and repeated two times. The relative growth rate and shoot regeneration percentage was calculated. After a month the relative growth rate of callus was calculated as the (FMf - FMi)/FMi. FMf is final fresh masse and FMi is initial fresh mass (Errabii et al., 2006).

### 2.4 Determination of free proline content

Determination of proline content derived from the leaves of potato (*Solunum tuberosum*) plants with sodium chloride by measuring the amount of color obtained from the reaction of proline with ninhydric acid (Mc Mannus et al., 2000) was performed in this study. We used a SmartSpec™ 3000 Bio-Rad spectrophotometer at 518 nm and proline concentration was determined from a standard curve and calculated on the basis of fresh weight.

### 2.5 Na^+^ and K^+^ analysis

Leaf and callus tissues were dried at 60 °C for at least 5 days, 10 ml of aqueous sulfosalicylic acid at 3% concentration are applied to 0.01 g of dried callus and leaf powder for 24 h at 4 °C, then the extracted sample is purified with Whatman No. 1 filter paperandthe dried ground leaf tissues (200 mg) were added to 5 ml HNO3, and the solution was filtered through Whatman filter paper 42 to final volume of 50 ml. Sodium and potassium content were measured by flame photometer following the procedure of Skooget al. (2007).

### 2.6 Statistical analysis

Statistical analysis of all experiments was completely randomized design with four replicates. Statistical analysis using technique of variance analysis (ANOVA) by multiple range tests is used to analyze the recorded data (Steel et al. 1997). The means significance was compared by applying the (L.S.D.) and the test is done at a 5% probability level.

## 3 Results

### 3.1 Callus induction and *in vitro* shoot regeneration

**Photo 1:**
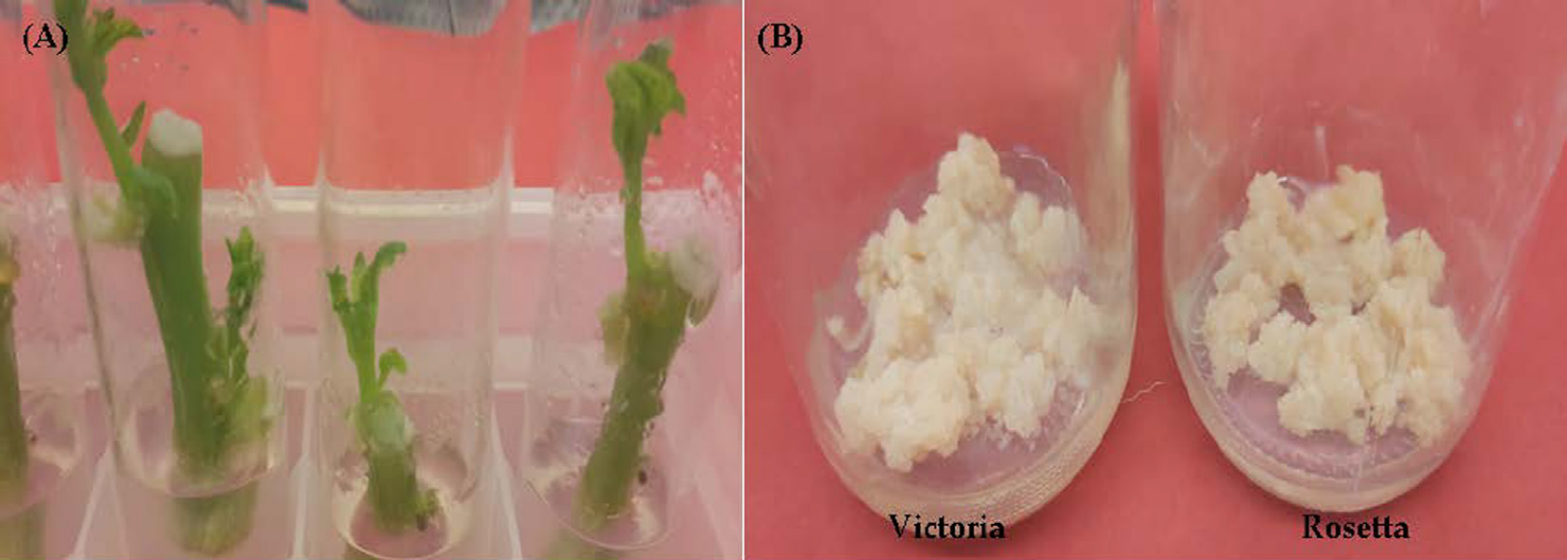
Leaf explants were excised from 3 to 4 week-*in vitro*-grown shoots of potato (*Solanumtuberosum*L.) cultivars Victoria and Rosetta and was cultured on MS medium containing0.1 mg l^−1^ BAP, 0.05 mg l^−1^ IBA and 1.0 mg l^-1^PBZ for callus induction (**A**). Callus formed on MS medium supplemented with 2.0 mg l^−1^ 2,4-D and 0.5 mg l^−1^ Kin (**B**).

In the present study, the complete regeneration was successfully achieved using *in vitro* leaf explants of potato (*Solunum tuberosum*) cultivars Victoria and Rosetta through callus culture (Photo 1 A). Leaf explants induced callus on MS medium accompanied with dissimilar applications of 2, 4-D + kinetin or NAA + BAP as shown in Table 1. The result showed that, the highest callus formation percentage was 83.50% for Rosetta cultivar and 93.40% for Victoria cultivar on MS medium including 1.5 and 2.0 mg l^−1^ 2, 4-D with 0.5 mg l^-1^kinetin, respectively (Photo 1 B). While, MS medium supplemented with 3.0 mg l^−1^ NAA with 0.5 and 1.0 mg l^-1^BAP was recorded (73.50 and 78.00%) of callus formation percentage for cvs. Rosetta and Victoria, respectively comparing with other treatments. It is worth noting that, these hormone combinations were the best in recording highest values for both RWC and calls formation. Also, at 1.5 or 2.5 mg l^−1^ 2,4-D with 0.5 mg l^−1^ kinetin the optimum rate (++++) of callus formation degree was recorded of both cultivars in addition to 3.0 mg l^−1^ NAA with 0.5 mg l^-1^BAP for cv. Rosetta only (Table 1). In contrast, combination 0.5 mg l^−1^ NAA with 0.5 mg l^-1^BAP recorded minimum values of callus induction percentage (17.0%; 12.5%) and RWC (16.35%; 11.78%) in cvs. Victoria and Rosetta, respectively. It is noted that no shoot regeneration growths were recorded either when 2–4-D and NAA applied alone at 0.5 or 3.0 mg l^−1^ and 0.5 or 5.0 mg l^−1^, respectively.

**Table 1:** Influence of plant growth regulators concentrations on callus formation percentage, relative water content (%) and degree of regeneration of potato (*Solunum tuberosum*) cvs. Victoria and Rosetta *via in vitro* leaf explants after 4 weeks.

Growth regulators (mg l-1)		% of Callus formation		Relative water content (RWC)		Degree of callus formation	
2,4-D	Kinetin	cv. Rosetta	cv. Victoria	cv. Victoria	cv. Rosetta	cv. Victoria	cv. Rosetta
0.5	0.0	42.50 ± 0.55f	38.75 ± 0.13g	40.25 ± 0.28e	36.50 ± 0.69g	+	+
1.0	0.0	56.35 ± 0.88e	67.25 ± 0.83d	45.75 ± 0.38e	63.35 ± 0.42d	++	++
1.5	0.0	72.15 ± 0.87c	79.25 ± 0.45b	69.50 ± 0.66d	73.15 ± 0.55c	++++	+++
2.0	0.0	78.25 ± 0.35b	72.30 ± 0.92c	72.45 ± 0.29c	69.25 ± 0.49d	++++	+++
2.5	0.0	68.75 ± 0.40d	61.45 ± 0.27d	61.25 ± 0.50d	49.50 ± 0.33e	++	+++
3.0	0.0	58.50 ± 0.22e	53.50 ± 0.77e	46.25 ± 0.91e	44.75 ± 0.30f	++	++
0.5	0.5	51.50 ± 0.55e	58.25 ± 0.51e	49.25 ± 0.66e	52.00 ± 0.18e	++	++
1.0	0.5	61.75 ± 0.35d	71.50 ± 0.92c	58.25 ± 0.35e	65.00 ± 0.33d	+++	++
**1.5**	**0.5**	78.25 ± 0.49b	83.50 ± 0.14a	63.75 ± 0.28d	78.25 ± 0.26b	++++	++++
**2.0**	**0.5**	93.40 ± 0.84a	78.00 ± 0.88b	83.70 ± 0.41a	71.00 ± 0.55c	++++	++++
2.5	0.5	80.25 ± 0.75b	69.25 ± 0.39d	74.25 ± 0.44c	63.50 ± 0.39d	+++	++++
3.0	0.5	63.25 ± 0.22d	60.00 ± 0.54d	59.00 ± 0.15e	56.50 ± 0.62e	++	++

**NAA**	**BAP**						
0.5	0.5	17.00 ± 0.22j	12.50 ± 0.77k	16.35 ± 0.53j	11.78 ± 0.77k	+	+
1.0	0.5	22.00 ± 0.15h	20.25 ± 0.52i	20.50 ± 0.55i	18.40 ± 0.15j	+	+
2.0	0.5	30.25 ± 0.65g	27.25 ± 0.93h	28.92 ± 0.16g	25.00 ± 0.73h	+	+
**3.0**	**0.5**	71.50 ± 0.29c	73.50 ± 0.48c	69.40 ± 0.28d	70.80 ± 0.39c	++++	+++
4.0	0.5	69.50 ± 0.81d	72.00 ± 0.52c	63.25 ± 0.71d	68.35 ± 0.42d	+++	+++
5.0	0.5	61.00 ± 0.44d	66.25 ± 0.65d	58.15 ± 0.14e	62.25 ± 0.64d	++	++
0.5	1.0	20.50 ± 0.25i	18.75 ± 0.33j	18.75 ± 0.59j	16.20 ± 0.26j	+	+
1.0	1.0	27.50 ± 0.39h	24.35 ± 0.39h	22.45 ± 0.48h	21.50 ± 0.19i	+	+
2.0	1.0	32.35 ± 0.24g	29.00 ± 0.25g	27.50 ± 0.50g	23.40 ± 0.47h	+	+
**3.0**	**1.0**	78.00 ± 0.88b	69.25 ± 0.66d	75.00 ± 0.17b	60.15 ± 0.35d	+++	++++
4.0	1.0	73.25 ± 0.94c	71.50 ± 0.23c	70.05 ± 0.30c	67.00 ± 0.59d	+++	+++
5.0	1.0	63.45 ± 0.15d	67.00 ± 0.19d	57.50 ± 0.95e	60.75 ± 0.36d	++	++

Values are means ± standard error of three replicates from two experiments. For each cultivar, bars with the same letters are not significantly different at P ≤ 0.05 level. Degree of calls formation are visually estimated assmall callus (+); moderate callus (++); massive callus (+++); quite massive callus (++++).

**Photo 2:**
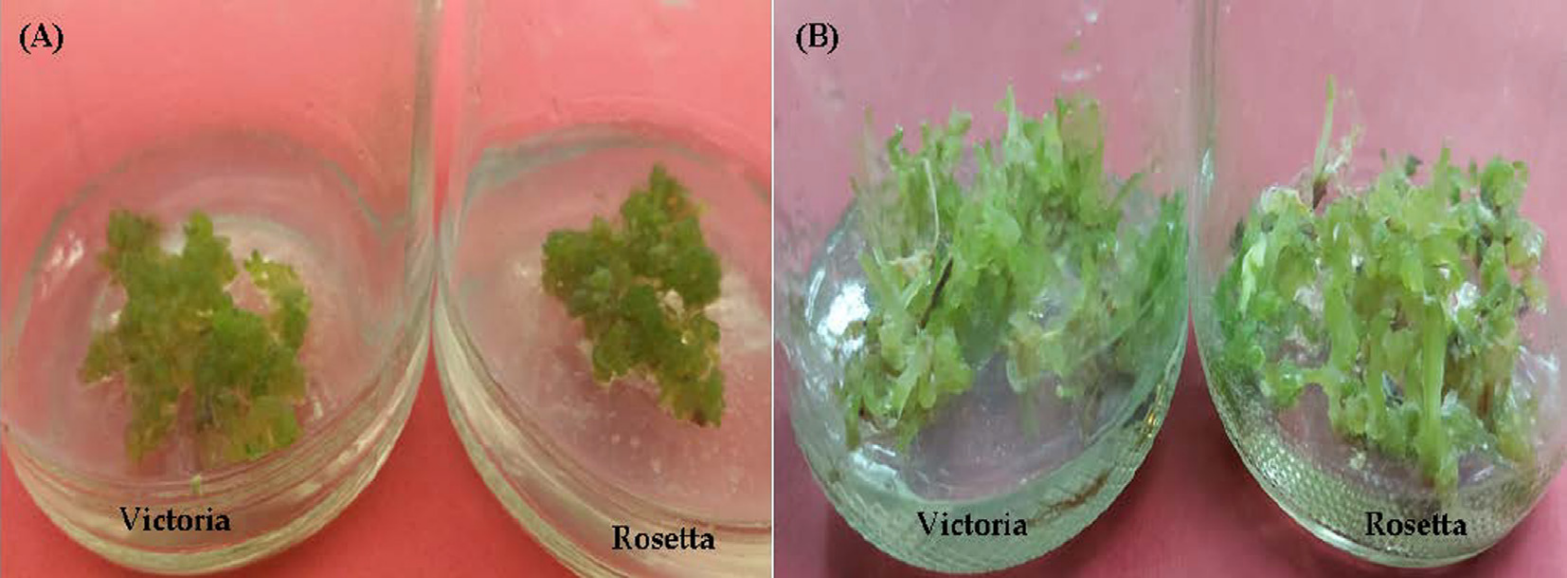
*In vitro* shoot regeneration was recorded from callusof potato (*Solanumtuberosum*L.) cultivars Victoria and Rosetta. (**A**) Matured somatic embryos derived from callus cultured on MS medium containing with 4.0 mg l^−1^ BAP and 5.0 mg l^−1^ Kin. **(B)** Adventitious shoot onMS medium containing 4.0 mg l^−1^ TDZ and 5.0 mg l^−1^ GA3after 4 weeks.

The callus obtained from *in vitro* leaf was transferred to the regeneration media containing 0.5 or 5.0 mg l^−1^ of BAP individual or with 0.5 mg l^−1^ kinetin and 1.0 or 5.0 mg l^−1^ TDZ individual or with 0.5 mg l^−1^ GA3. Observation of shoot regeneration was recorded from callus as shown in Table 2. The result showed that, the highest percentage of shoot regeneration (87% and 89.5%) was showed on MS medium supplemented with 4.0 or 5.0 mg l^−1^ TDZ with 5.0 mg l^−1^ GA3 in Rosetta and Victoria, respectively (Photo 2). The highest mean number of shoots (4.92) and mean shoot length (2.52 cm) for cv. Victoria occurred on 4 mg l^−1^ TDZ and 0.5 mg l^−1^ GA3. While, the highest mean numbers of shoots (4.85) induced from callus and mean of shoot length (2.49 cm) for cv. Rosetta on 5 mg l^−1^ TDZ + 0.5 mg l^−1^ GA3. The results indicated that the addition of BA individual at 0.5 or 1.0 at mg l^−1^ scored zero value for all the studied traits while the TDZ succeeded in being effective in the in case of using it alone (Table 2).

**Table 2:** Effect of BAP and TDZ alone or in combination with kinetin and GA_3_ in MS medium on shoot regeneration from leaf derived callus of potato (*Solunum tuberosum*) cvs. Victoria and Rosetta after five weeks.

**Growth regulators (mg 1**–**1)**		**% of shoot regeneration**		**Mean number of shoots/callus**		**Mean shoot length (cm)**	
**BAP**	**Kinetin**	**cv.Victoria**	**cv.Rosetta**	**cv.Victoria**	**cv.Rosetta**	**cv.Victoria**	**cv.Rosetta**
0.5	0.0	00.0 ± 0.00i	00.0 ± 0.00i	00.0 ± 0.00h	00.0 ± 0.00h	00.0 ± 0.00h	00.0 ± 0.00h
1.0	0.0	00.0 ± 0.00i	00.0 ± 0.00i	00.0 ± 0.00h	00.0 ± 0.00h	00.0 ± 0.00h	00.0 ± 0.00h
2.0	0.0	15.0 ± 0.43h	13.5 ± 0.13h	0.85 ± 0.66g	0.78 ± 0.43g	0.75 ± 0.59f	0.55 ± 0.25g
3.0	0.0	35.0 ± 0.55f	28.0 ± 0.17g	1.25 ± 0.41e	1.05 ± 0.75f	1.00 ± 0.72e	0.95 ± 0.22f
4.0	0.0	57.5 ± 0.76d	48.5 ± 0.36e	1.85 ± 0.27e	1.25 ± 0.26e	1.40 ± 0.33d	1.35 ± 0.39d
5.0	0.0	64.5 ± 0.54c	60.0 ± 0.77d	2.25 ± 0.26d	1.38 ± 0.44e	1.25 ± 0.92d	1.05 ± 0.31e
0.5	0.5	00.0 ± 0.00i	00.0 ± 0.00i	00.0 ± 0.00h	00.0 ± 0.00h	00.0 ± 0.00h	00.0 ± 0.00h
1.0	0.5	17.5 ± 0.29h	12.0 ± 0.15h	1.25 ± 0.18e	0.98 ± 0.80g	1.00 ± 0.29e	1.18 ± 0.82e
2.0	0.5	34.5 ± 0.81f	30.0 ± 0.22f	1.73 ± 0.22e	1.05 ± 0.57f	1.32 ± 0.48d	1.45 ± 0.77d
**3.0**	**0.5**	55.0 ± 0.49d	69.5 ± 0.27d	2.62 ± 0.35d	2.72 ± 0.71d	1.55 ± 0.43c	1.75 ± 0.12c
**4.0**	**0.5**	78.5 ± 0.33b	67.5 ± 0.19d	3.25 ± 0.52c	1.45 ± 0.50e	1.85 ± 0.26c	1.62 ± 0.40c
5.0	0.5	69.0 ± 0.89c	62.0 ± 0.45d	2.75 ± 0.49d	1.28 ± 0.11e	1.05 ± 0.79e	1.00 ± 0.64e

**TDZ**	**GA3**						
1.0	0.0	22.5 ± 0.42g	21.0 ± 0.29g	2.25 ± 0.18d	1.60 ± 0.38e	0.78 ± 0.19f	0.58 ± 0.54g
2.0	0.0	45.5 ± 0.39e	40.0 ± 0.35e	2.50 ± 0.26d	2.25 ± 0.88d	0.98 ± 0.88f	0.75 ± 0.23f
3.0	0.0	66.0 ± 0.55c	64.0 ± 0.43d	3.72 ± 0.55c	3.60 ± 0.37c	1.25 ± 0.37d	1.05 ± 0.76e
4.0	0.0	84.5 ± 0.69a	70.0 ± 0.66c	4.00 ± 0.29b	3.95 ± 0.11c	1.55 ± 0.40c	1.25 ± 0.55d
5.0	0.0	72.0 ± 0.83b	74.5 ± 0.28b	3.85 ± 0.43c	3.78 ± 0.62c	1.39 ± 0.85d	1.48 ± 0.39c
1.0	0.5	38.0 ± 0.22f	28.0 ± 0.57g	2.35 ± 0.32d	1.75 ± 0.44e	2.15 ± 0.25b	1.94 ± 0.77c
2.0	0.5	52.0 ± 0.59d	48.0 ± 0.46e	2.75 ± 0.74d	2.35 ± 0.90d	2.32 ± 0.44b	1.99 ± 0.42c
3.0	0.5	74.0 ± 0.38b	70.0 ± 0.34c	3.95 ± 0.42c	3.75 ± 0.71c	2.45 ± 0.61a	2.08 ± 0.13b
**4.0**	**0.5**	89.5 ± 0.26a	75.0 ± 0.93b	4.92 ± 0.51a	4.62 ± 0.47a	2.52 ± 0.37a	2.30 ± 0.55b
**5.0**	**0.5**	78.0 ± 0.18b	87.0 ± 0.79a	4.60 ± 0.30a	4.85 ± 0.58a	2.18 ± 0.77b	2.49 ± 0.36a

Values are means ± standard error of three replicates from two experiments. For each cultivar, bars with the same letters are not significantly different at P ≤ 0.05 level

### 3.2 *In vitro* production of microtubers

Plantlets were used as explants to produce *in vitro* microtubers in culture media containing half strength (MS basal media) using various levels of sucrose (50; 60; 70 ;80 and 90 g/l) with different concentrations of KIN (mg/l) and their combinations on *in vitro* microtubrization for both tested potato cultivars are illustrated in Table 3. The highest percentage of microtuber per explant (92.0 and 99.0%) was observed on MS medium supplemented with 3.0 mg l^−1^ kinetin and 1.0 mg l^-1^paclobutrazol (PBZ) with 80 g l^−1^ sucrose after two months of Rosetta cultivar and 90 g l^−1^ sucrose of Victoria cultivar, respectively. While, the highest number of microtuber/explant (5.45 and 5.80) was observed on the same medium for both Rosetta and Victoria cultivars, respectively.

**Table 3:** The effect of different levels of sucrose and the best concentrations of kinetin *with* 1.0 mg l^-1^PBZon the microtubrization stage of potato (*Solunum tuberosum*) cvs. Victoria and Rosetta after two months**.**

**Kin (mg l^−1^)**									
	**Tr.**	**0.0**		**2.0**		**3.0**		**5.0**	
**Parameters**	**Sucrose (g l^−1^)**	**Victoria**	**Rosetta**	**Victoria**	**Rosetta**	**Victoria**	**Rosetta**	**Victoria**	**Rosetta**
**% of explant formed microtuber**	50	16.0 ± 0.50k	14.0 ± 0.32k	22.0 ± 0.19j	20.0 ± 0.25j	37.0 ± 0.66i	42.0 ± 0.44h	38.0 ± 0.19i	32.0 ± 0.55i
	60	28.0 ± 0.22j	25.0 ± 0.18j	38.5 ± 0.49i	32.5 ± 0.47i	55.0 ± 0.32g	72.5 ± 0.22e	52.0 ± 0.15g	50.0 ± 0.28g
	70	54.0 ± 0.43g	51.0 ± 0.33g	68.0 ± 0.77f	63.0 ± 0.61f	72.0 ± 0.55e	81.5 ± 0.56d	70.0 ± 0.31e	77.0 ± 0.33d
	80	64.5 ± 0.28f	62.0 ± 0.54f	79.0 ± 0.92d	71.0 ± 0.39e	87.5 ± 0.87c	92.0 ± 0.77b	80.0 ± 0.40d	83.0 ± 0.76c
	90	73.0 ± 0.84e	71.5 ± 0.66e	86.5 ± 0.55c	83.0 ± 0.63d	99.0 ± 0.49a	89.0 ± 0.45b	91.0 ± 0.88b	79.5 ± 0.47d
**No. of microtuber/explant**	50	1.95 ± 0.24k	1.52 ± 0.19k	2.20 ± 0.25i	2.05 ± 0.55i	2.80 ± 0.26h	2.50 ± 0.33h	2.65 ± 0.53h	2.35 ± 0.33i
	60	2.60 ± 0.55h	2.40 ± 0.33h	3.65 ± 0.61f	3.45 ± 0.83g	3.75 ± 0.35f	3.55 ± 0.15f	3.25 ± 0.44g	3.00 ± 0.26g
	70	2.75 ± 0.39h	2.50 ± 0.42h	4.00 ± 0.43e	3.85 ± 0.60f	4.70 ± 0.91c	4.87 ± 0.73c	4.60 ± 0.16d	4.50 ± 0.85d
	80	3.40 ± 0.52g	3.25 ± 0.65g	4.35 ± 0.77e	4.05 ± 0.85e	4.95 ± 0.66c	5.45 ± 0.38a	4.82 ± 0.62c	4.75 ± 0.77c
	90	3.87 ± 0.66f	3.65 ± 0.72f	4.90 ± 0.49c	4.50 ± 0.77d	5.80 ± 0.46a	5.20 ± 0.84b	5.25 ± 0.55b	4.98 ± 0.22c
**Microtuber weight/explant (mg)**	50	255 ± 0.50h	230 ± 0.75h	380 ± 0.19g	368 ± 0.26g	437 ± 0.25f	470 ± 0.15e	390 ± 0.44g	378 ± 0.62g
	60	362 ± 0.35g	360 ± 0.44g	389 ± 0.22g	378 ± 0.33g	480 ± 0.39e	499 ± 0.26e	405 ± 0.72f	415 ± 0.39f
	70	480 ± 0.38e	369 ± 0.38g	498 ± 0.71e	470 ± 0.39e	525 ± 0.41d	560 ± 0.85c	518 ± 0.17d	509 ± 0.48d
	80	495 ± 0.59e	472 ± 0.65e	528 ± 0.50d	515 ± 0.46d	615 ± 0.88b	675 ± 0.46a	548 ± 0.54d	535 ± 0.59d
	90	522 ± 0.40d	505 ± 0.78d	568 ± 0.49c	550 ± 0.29c	690 ± 0.60a	635 ± 0.80b	579 ± 0.61c	575 ± 0.73c

Values are means ± standard error of three replicates from two experiments. For each cultivar, bars with the same letters are not significantly different at P ≤ 0.05 level

### 3.3 Callus proliferation and plant regeneration response to NaCl stress

**Fig. 1:**
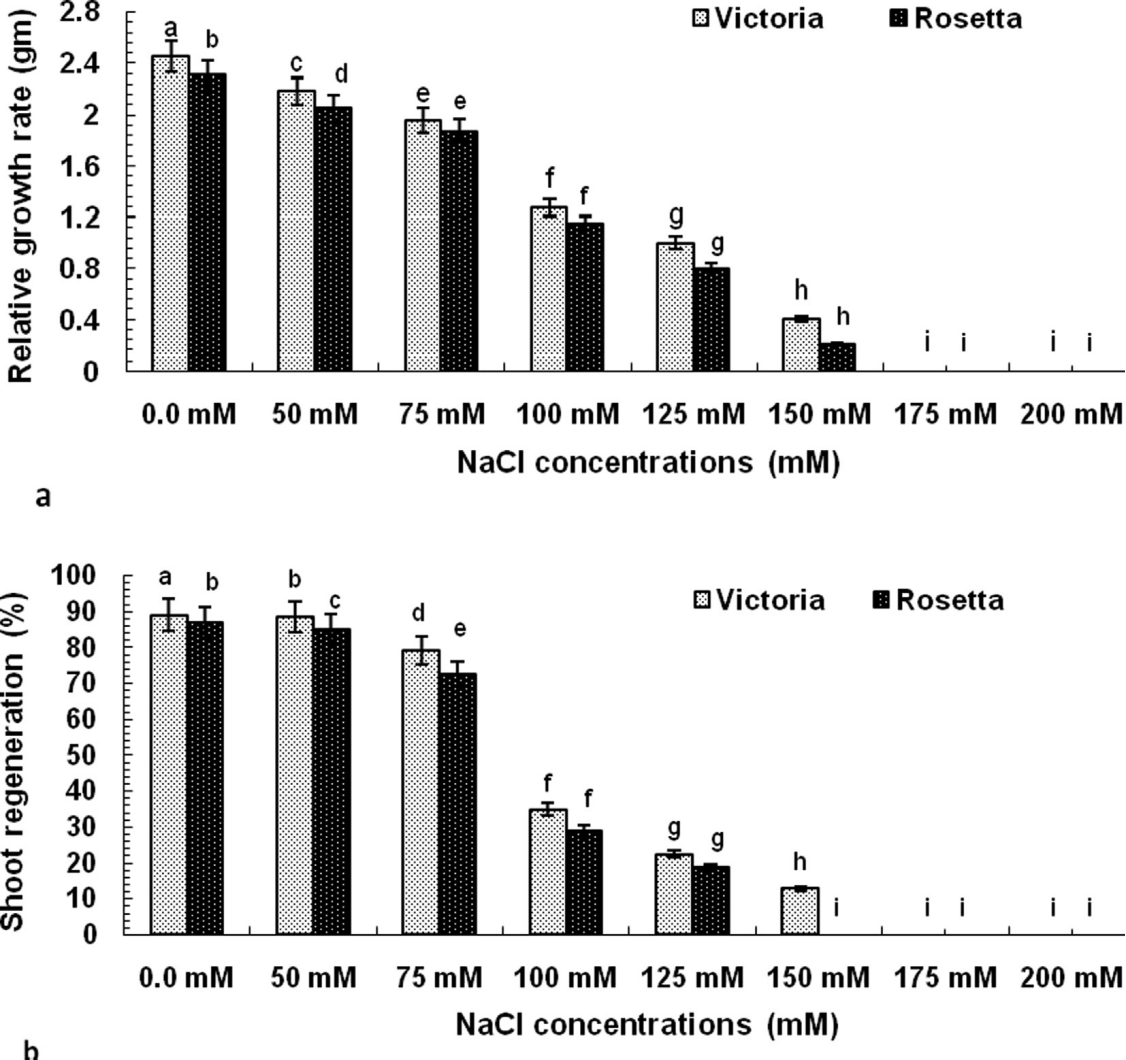
Effect of different NaCl concentrations on the **a**) relative growth rate of callus after one month and **b**) shoot regeneration percentage of potato (*Solunum tuberosum*) cvs. Victoria and Rosetta.

**Photo 3:**
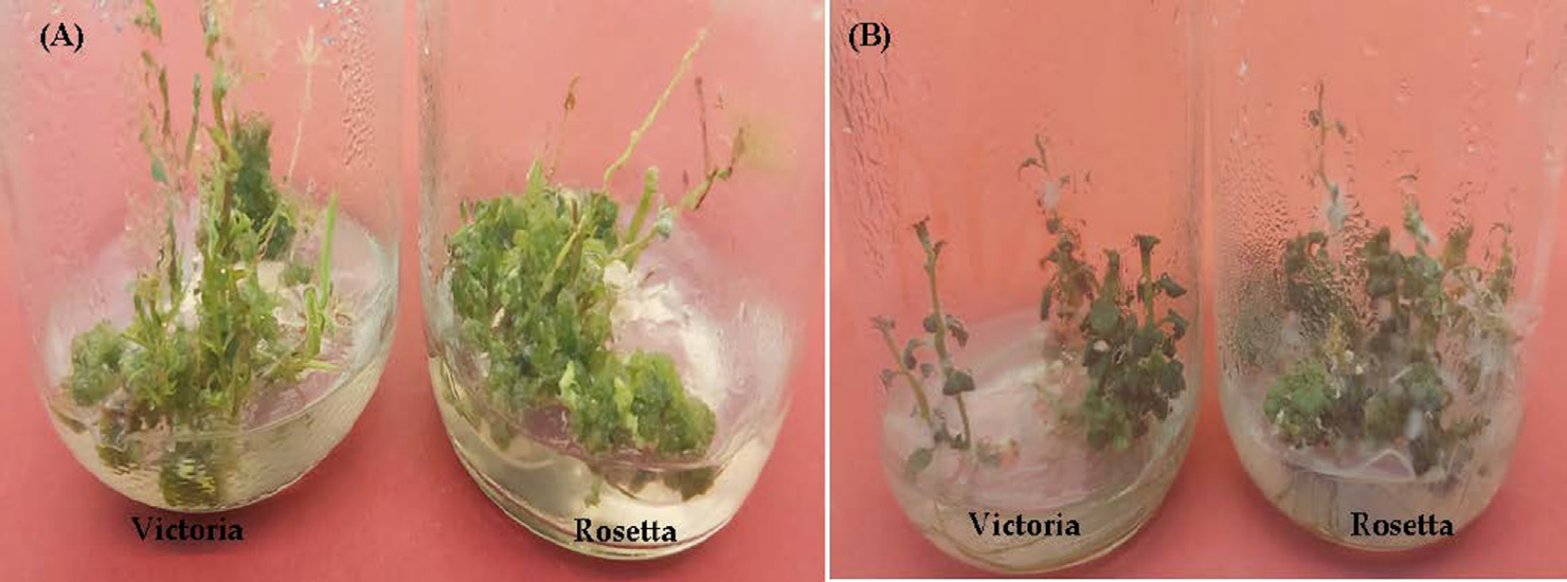
Shoot regeneration from callus of potato (*Solunumtuberosum*) under salt stress.Shoot formed on MS medium containing5.0 mg l^−1^ TDZ and 5.0 mg l^−1^ GA3 under 125 mMNaCl (**A**) plantlets formed MS medium containing3.0 mg l^-1^Kin and 1.0 mg l^−1^ PBZunder 125 mMNaCl (**B**).

**Photo 4:**
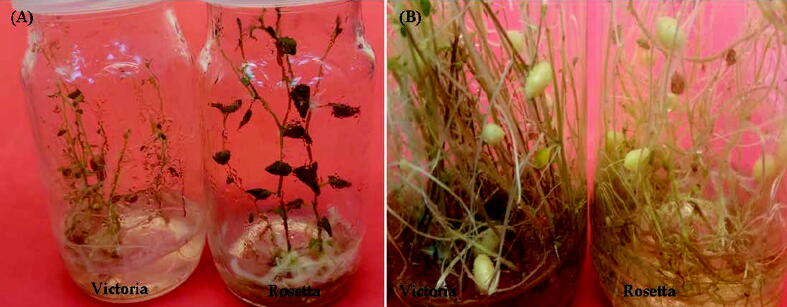
*In vitro* production of microtubers on MS medium supplemented with 3.0 mg l^−1^ Kin and 1.0 mg l^−1^ PBZ with 90 g l^−1^ sucrose under 125 mMNaCl of potato (*Solanumtuberosum*L.) cultivars Victoria and Rosetta after one months (**A**) and after two months **(B).**

NaCl-tolerant calli were obtained by gradual selection to evaluate the effect of NaCl on different parameters as mentioned in materials and methods section. The gradual selection process resulted in the establishment of NaCl-tolerant callus lines showing a good cell proliferation, in spite of callus growth rate decreased with the increase of salt concentration on culture medium. All callus tissue did not possess the ability to grow under concentrations of salts beginning from 175 and 200 mM of cv. Victoria and 150 mM of cv. Rosetta (Fig. 1 and Photo 3) (see Photo 4).

### 3.4 Propagation of microtuber potato seeds under salt stress

**Fig. 2:**
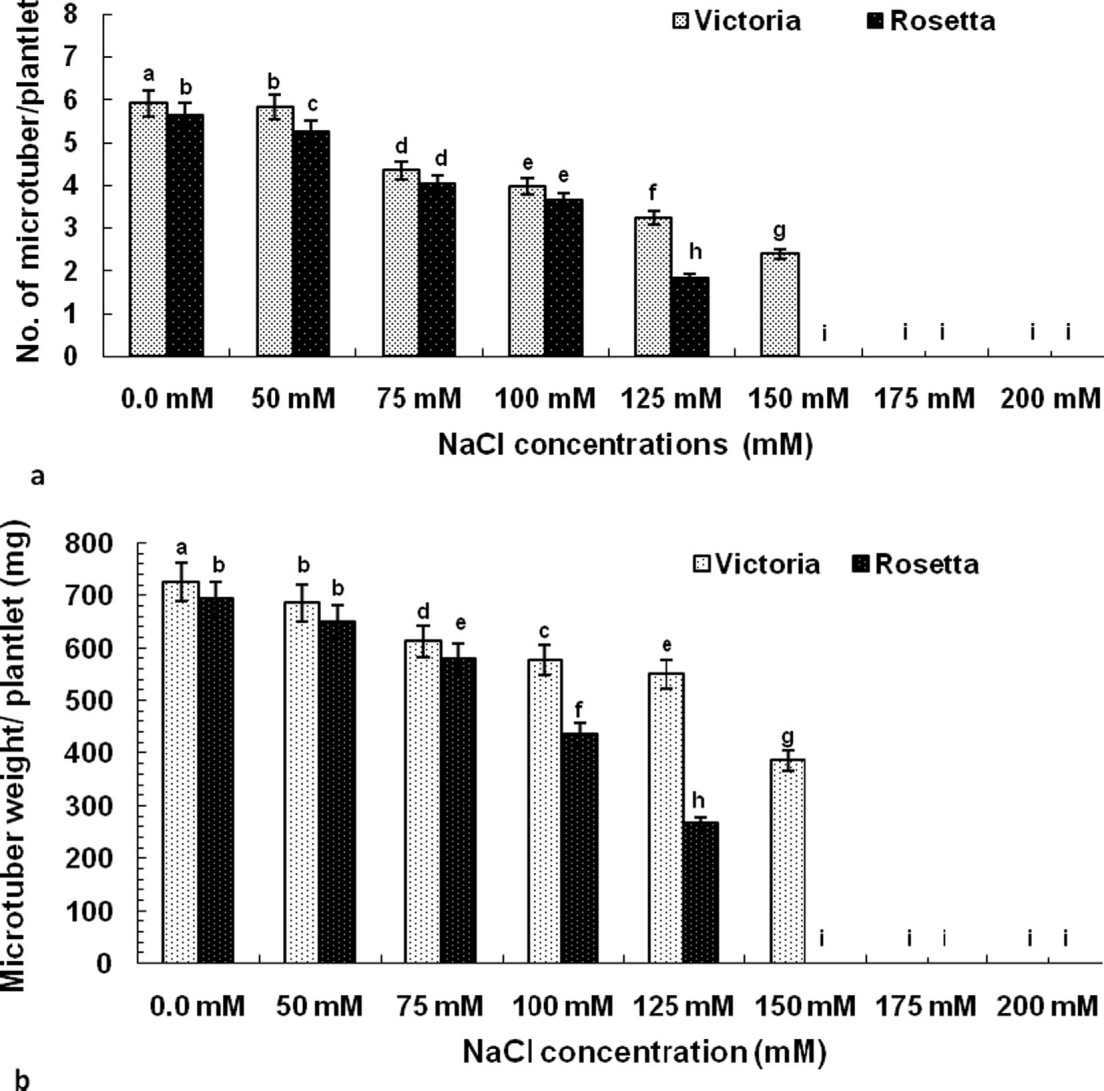
The effect of different NaCl concentrations on the number of microtuber/plantlet (**a**) and microtuber weight/plantlet (**b**) of potato (*Solunum tuberosum*) cvs. Victoria and Rosetta were cultured on MS medium supplemented with 3.0 mg l-1 kin, 1.0 mg l-1PBZ and 90 g l-1 sucrose.

The effect of different NaCl concentrations on the number of microtuber per plantlet and microtuber weight per plantlet (mg) of potato cultivars Victoria and Rosetta were cultured on MS medium supplemented with 3.0 mg l^−1^ kinetin, 1.0 mg l^-1^paclobutrazol (PBZ) and 90 g l^−1^ sucrose as shown in Table 4 and Fig. 2 (See Photo 4).

**Table 4:** Sodium and potassium content in callus of potato (*Solunum tuberosum*) cvs. Victoria and Rosetta subjected to different concentrations of NaCl.

**NaClconcentrations (mM)**	**K + content (mg.g–1 DW)**		**Na + content (m.g–1 DW)**		**Na+/K+ index**	
	**Victoria**	**Rosetta**	**Victoria**	**Rosetta**	**Victoria**	**Rosetta**
0.0	11.45 ± 0.18a	10.85 ± 0.28b	2.25 ± 0.48f	2.05 ± 0.72f	0.20 ± 0.28i	0.19 ± 0.37i
50	9.75 ± 0.59c	10.05 ± 0.47b	15.90 ± 0.33e	15.42 ± 0.56e	1.63 ± 0.50h	1.53 ± 0.49h
75	9.18 ± 0.74c	9.15 ± 0.55c	36.40 ± 0.19e	38.90 ± 0.39e	3.97 ± 0.35g	4.25 ± 0.33f
100	7.90 ± 0.38d	7.78 ± 0.25d	44.35 ± 0.59d	49.25 ± 0.88d	5.61 ± 0.76e	6.33 ± 0.65d
125	5.60 ± 0.39e	5.25 ± 0.48e	59.25 ± 0.38c	62.73 ± 0.29a	10.58 ± 0.49c	11.95 ± 0.88b
150	3.45 ± 0.42f	00.00 ± 0.00g	61.50 ± 0.65b	00.00 ± 0.00g	17.83 ± 0.72a	00.00 ± 0.00j
175	00.00 ± 0.00g	00.00 ± 0.00g	00.00 ± 0.00g	00.00 ± 0.00g	00.00 ± 0.00j	00.00 ± 0.00j
200	00.00 ± 0.00g	00.00 ± 0.00g	00.00 ± 0.00g	00.00 ± 0.00g	00.00 ± 0.00j	00.00 ± 0.00j

Values are means ± standard error of three replicates from two experiments. For each cultivar, bars with the same letters are not significantly different at P ≤ 0.05 level.

### 3.5 Sodium and potassium ions content

The results showed that the sodium content of callus and leaves increased with increasing concentrations of NaCl in the medium, and the level of potassium in callus and leaves decreased significantly in both cultivars (Table 5). The Na+ content in the treatments increased between 30 times with regard to 0.0 mMNaCl of both cultivars. The highest content of Na+ (60.00 and 62.7 mM) and lowest content of K+ (4.70 and 5.25 mM) were detected under NaCl at 125 mM for cvs. Victoria and Rosetta in case of leaves and callus, respectively. Regarding the Na^+^/K^+^ content, it was found that, it recorded the highest values in the cv. Victoria comparing to cv. Rosetta in the case of leaves, while it was fluctuating between the two cultivars in the case of callus (Table 2). The highest percentage of Na^+^/K^+^ (17.83 and 25.67) was recorded in the 150 mM treatment of the cv. Victoria in the case of leaves and calluses respectively.

**Table 5:** Sodium and potassium content in leaves of potato (*Solunum tuberosum*) cvs. Victoria and Rosetta subjected to different concentrations of NaCl.

**NaClconcentrations (mM)**	**K + content (mg g–1 DW)**		**Na + content (m g–1 DW)**		**Na+/K+ index**	
	**Victoria**	**Rosetta**	**Victoria**	**Rosetta**	**Victoria**	**Rosetta**
0.14 ± 0.24h	0.17 ± 0.29h	1.87 ± 0.56j	2.98 ± 0.38i	13.62 ± 0.59b	17.65 ± 0.45a	0.0
1.27 ± 0.32g	1.66 ± 0.45f	12.35 ± 0.25h	17.35 ± 0.24g	9.76 ± 0.44d	10.84 ± 0.82c	50
3.73 ± 0.14e	4.02 ± 0.71d	33.50 ± 0.44f	41.25 ± 0.55e	8.98 ± 0.62e	10.25 ± 0.38c	75
6.40 ± 0.66c	6.11 ± 0.91c	42.58 ± 0.39e	49.70 ± 0.39d	6.65 ± 0.72f	8.14 ± 0.25e	100
12.20 ± 0.35c	12.63 ± 0.85b	57.35 ± 0.19c	60.00 ± 0.48b	4.70 ± 0.49g	4.75 ± 0.75g	125
00.00 ± 0.00i	25.67 ± 0.72a	00.00 ± 0.00k	68.80 ± 0.68a	00.00 ± 0.00i	2.68 ± 0.59h	150
00.00 ± 0.00i	00.00 ± 0.00i	00.00 ± 0.00k	00.00 ± 0.00k	00.00 ± 0.00i	00.00 ± 0.00i	175
00.00 ± 0.00i	00.00 ± 0.00i	00.00 ± 0.00k	00.00 ± 0.00k	00.00 ± 0.00i	00.00 ± 0.00i	200

Values are means ± standard error of three replicates from two experiments. For each cultivar, bars with the same letters are not significantly different at P ≤ 0.05 level

### 3.6 Proline content

**Fig. 3:**
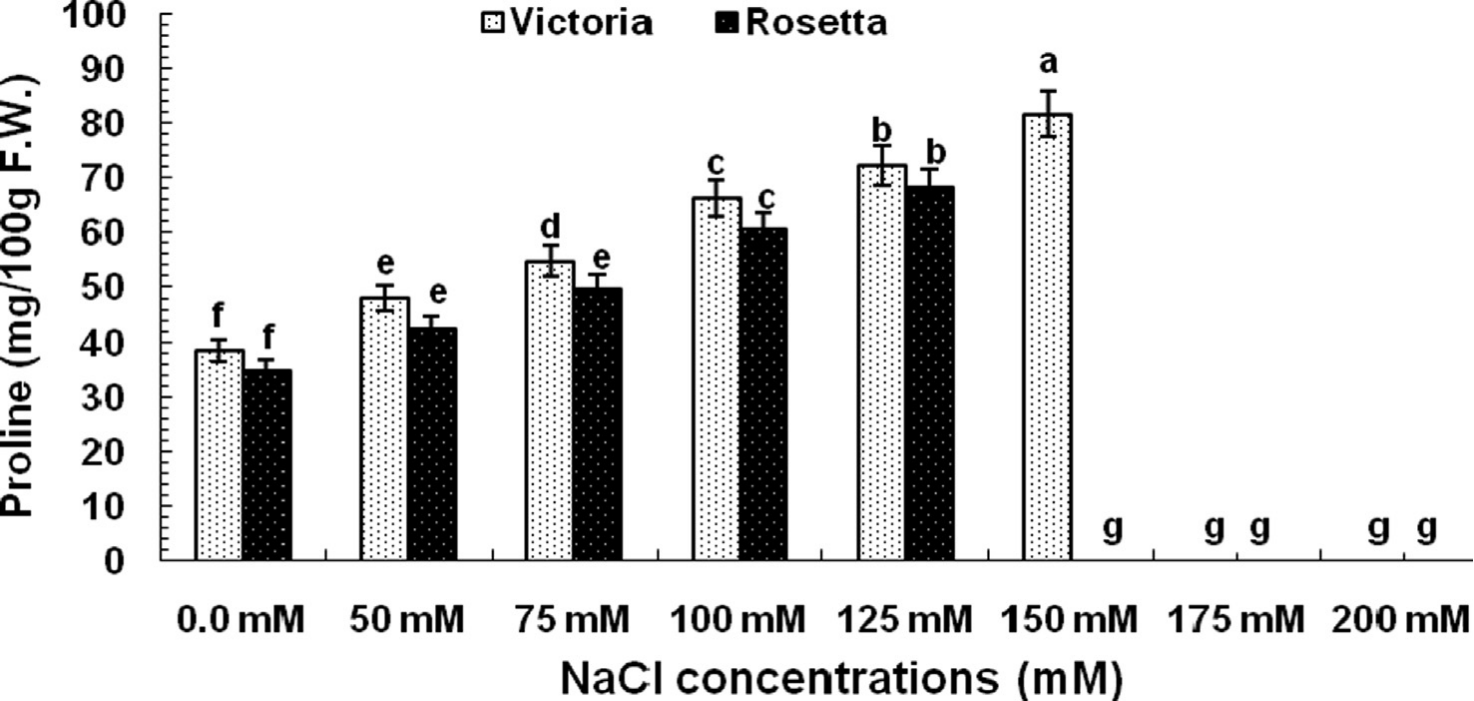
Effect of different NaCl concentrations on proline content (mg/100 g fresh weight) in potato (*Solunum tuberosum*) cvs. Victoria and Rosetta.

The general pattern of proline in leaves of two cultivars was relatively increased as salt concentration increased (Fig. 3). The highest amount of proline (81.7 mg/100 g F.W.) was observed at 150mMNaCl of Victoria cultivar. However, the highest amount of proline (68.3 mg/100gF.W.) was observed at 125 mMNaCl in leaf of Rosetta cultivar compared with other treatments. While, the amounts of proline in leave of non-treated plantlets were less than treated plantlets.

## 4 Discussion

The success in finding a protocol for the regeneration of potato plants by using leaf explant *in vitro* depends on a variety of factors, the most important of which are cultivars and combinations of PGRs following by composition of artificial medium, type of explants, season, plant physiology and growth conditions under growth chamber (Abeuova et al., 2020). Consequently, this study is concerned with finding the most appropriate PGRs combinations to obtain a high percentage of plant regeneration indirectly through the tissues of callus and in the presence of two variables; the plant variety used and combination of PGRs. The results showed that the values of callus induction %, RWC and degree of callus formation are varied between the two used cultivars under study, also, the application of PGRs affected the callogenesis (Table 1). Application of 2,4-D combined with kin at (1.5 + 0.5) mg l^-1^ and (2.0 + 0.5) mg l^−1^ are the best doses comparing to other treatments to enhance the callus induction %, RWC and degree of callus formation of cv. Victoria and cv. Rosetta, respectively. Our results are in agreement with studies demonstrating that 2, 4-D is an important plant hormone for callus induction (Metwali et al., 2020).

Also, the findings of Sané et al., 2012, Rathore et al., 2020 have confirmed the importance of genotype and the structure of the cultivation induction media over the callus induction. Compared to other auxins, hypotheses developed to explain the positive action of 2, 4-D that could be due to inhibition of the DRT102 protein, which is responsible for stimulating cell division and DNA replication. Hypotheses developed to explain the positive action of 2, 4-Duse compared to other auxins may be due to inhibiting the DRT102 protein, which is responsible for stimulating cell division, DNA replication (Pasternak et al., 2002) and lead to decrease in the number of cells. Moreover, the effect of 2, 4-D has been explained on the basis thatit acts as a signal cascade trigged to start a hyperpolarization of membrane polypeptides (Zuo et al., 2002). On the other hand, both potato cultivars maintained relatively high RWC (>50%) in most of the treatment with 2, 4-D and Kin comparing to treatment with NAA and BAP (Table 1). Etienne et al., 1991, Aldhebiani et al., 2018 mentioned that the water state in callus is an apparently important contributing factor for the initiation of somatic embryogenesis, and RWC has been identified as a suitable physiological marker of its embryogenic state.

The current study showed that the combination of TDZ + GA3 was superior to combination of BAP + Kinetin in encouragement to obtain high values for each ofshoot length, shoot regeneration and number of shoots/callus at all the treatments and significantly affecting these traits (P ≤ 0.05) (Table 2). Application of TDZ (4.0 mg l^−1^) + GA3 (0.5 mg l^−1^) was the optimal for obtaining high values of the previous traits. Such findings contradict previous studies which indicated the superiority of BA over TDZ for the axillary shoot multiplication of potato, in which no multiple shoots were observed on TDZ-supplemented medium (Mohamed et al., 2007) and a combination of BA and Kin as cytokines was reported as an enhanced reaction in state of shoots/explant, shoot length and no. of leaves in several potato cultivars comparing to TDZ (Kumlay and Ercisli, 2015, Mengs and M., Egigu, M., C., Abraha, E., 2018). The results of the present study are in agreement with Pérez-Tornero and Egea, AVanoostende, J., Burgos, L., 2000, Mutasim et al., 2010 who reported that the best results for regeneration percentages and proliferation of the shoot were obtained with thidiazuron (TDZ) instead of 6-benzylamino-purine (BAP). TDZ is considered one of the best growth regulators to obtain large number of potato plantlets and shoot induction of callus under stress conditions comparing with other growth regulators such as BA (Khalafalla et al., 2010). Also, previous study indicated the efficiency of TDZ to enhance Agrobacterium-mediated transformation in *Petunia hybrid* (Thirukkumaran et al., 2009). Several potato studies have employed BAP and NAA as growth regulators, however, cell regeneration efficiency was low under salt stress, so this hormone was used to induce cells to produce embryos and shoots. Because the higher the regeneration efficiency after incubation with bacteria, the better the efficiency of genetic transformation, the current work will be highly valuable in employing agrobacterium as a gene transfer method. In addition, TDZ alone or in combination with GA3 had significant difference at (P ≤ 0.05) in shoot regeneration, number of shoots/callus and shoot length **(**Table 2**).** The relatively broad spectrum role for GA3, at lower concentration, on cell enlargement and cell division was also reported in previous study (El Far, 2007), the author found that neither IAA, NAA, nor GA3 enhanced shoot proliferation in potato but GA3was the only hormone that best enhance shoot elongation. The action of GA3 on elongating cells may be attributed to the hypothesis that GA3 may works to increase the hydrolysis of starch to sugar, causing a decrease in the water potential inside the cells, which in turn helps to allow more water to enter the cell and increasing the plasticity of the cell walls, causing the elongation of the cells (Kumlay and Ercisli, 2015).

The variable response between cv. Victoria and cv. Rosetta are detected under most of the treatments (Table 2). Roodbar-shojaei et al. (2010), the authors documented that the responses of different cultivars to the same culture were different, because of differences of genotypes, and similarities were because of growth regulators and genotypes similarities. The hypothesis that explained the difference of interaction of different cultivars in a response to give the same effect to the studied traits with the unification of all cultivation conditions could be dueto variation in the rate of endogenous growth regulators of each cultivars (Schween and Schwenkel, 2003).

*In vitro* microtuberization in potato is influenced by several factors including PGRs, sucrose and genotype (Abeuova et al., 2020, Al-Hussaini et al., 2015, Mohapatra, 2018). This can be explained by the fact that sucrose is converted to starch during microtuber development and the accumulation of starch contributes to an increase in size and weight of microtubers. In the present work, various levels of sucrose (50; 60; 70; 80 and 90 g l^−1^) with different concentrations of Kin (0, 2, 3, 4 mg l^−1^) and their combinations were applied (Table 3). Our results showed that the percentage of explant formed microtuber, no. of microtuber / explant and microtubers weight / explants achieved the highest value on the media supplemented with Kin 3 mg l^−1^ and sucrose at 80 or 90 g l^−1^ in both cv Rosetta and cv. Victoria, respectively. Uranbey, 2005, Yagiz et al., 2020 defined that the degree of cell sensitivity towards the regulators, origin of explants and endogenous levels of PGRs are assumed to be the factors that determining the best concentration of Kin in culture media. On the other hand, Motallebi-Azar et al. (2013) stated that the high concentrations of sucrose, which are essential for induction of microtuber, serve as energy source *via* the osmotic effect. Visser et al. (1994) reported that when sucrose converts to starch, the coordination of genes expression due to starch and protein biosynthesis increases cell division and rapid expansion of stolon ends. According to Mashhad and Moeini, (2015), it was reported that the use of BAP and kinetin improved potato microtuber production by reducing the time required to start microtuberization, increasing the number of microtuber per explant and increase the weight and diameter of microtuber. This outcome is further confirmation for our results.

The selection of cultivars of potato, either *in vitro* or *in vivo*, under stresses conditions of salinity is often done in many experiments using NaCl, where Na+ and Cl ions are the most destructive elements exposed to salinity stress (Flowers et al., 2014). The presence of sodium chloride in the plant growth medium leads to often induces secondary stresses (Al Kharusi et al., 2017). The *in vitro* selection pattern for salt tolerant cultivars based on NaCl is less time consuming and allowing quick identification of tolerant cultivars (Mousavi et al., 2020). In the current study, screening of both cv Rosetta and cv. Victoria under different concentration of NaCl (0.0, 50, 75, 100, 125, 150, 175, 200 mM) was tested by measuring growth markers such as % of shoot regeneration, no. of microtuber/plantlet, microtuber weight/plantlet, RWC and biochemical marker such as Na+, K+, Na+/ K+, proline. There was a significant inhibition of NaCl on values of traits except Na+, Na^+^/K^+^,and proline (Table 4, Table 5 and Fig. 1, Fig. 2, Fig. 3). The growth of both cultivars was inhibited by increasing NaCl treatment, but cv Rosetta growth was completely stopped and died below 150 mMNaCl concentration. This effect may be attributed to NaCl, which inhibits the absorption of water and mineral elements by the roots, resulting in the lack of growth requirements of the plant and the appearance of symptoms based on the resistance of each plant (Munns, 2002). Furthermore, the results proved that the effect of salt treatment gave the same results, whether using callus or leaves cultures (Table 4, 5). Therefore, this research recommends selection at the cell level*in vitro*. In the current study increasing K^+^ values have a direct correlation with increased concentration of NaCl and cv. Victoria has a highest value of K^+^ compared with cv. Rosetta. This may be due to K +, which is an essential ionsthat control the electrolyte leakage and consequently maintains membrane integrity (Anschütz et al., 2014). In several investigations, Na^+^, K^+^ and K^+^/Na^+^ ratio are reported to be a physiological marker that determinate the salt stress tolerance (Craig and Moller, 2010, Horie and Schroeder, 2004).

To date, there is very little information on the effect of salinity on the development of potato microtuberand quality characteristics (Zhang et al., 2005). In the current study the mean number and fresh weight of microtubers in the two cultivars were also decreased significantly with an increase in saline levels. From the results, Fig. 2, it is inferred that the relationship between salinity levels with tuber number and tuber weight is an inverse relationship, suggesting that increased salt minerals level reduce the number of micro-tubers. The harmful effects of salinity on microtuberization of potato cultivars may have been explained in the previous studies as a result of: 1) reduction in osmotic potential (Pour et al., 2010); 2) reduction of water content and nutrient uptake (Silva et al., 2001). Ebadi and Iranbakhsh (2011) stated that the reduction in tuber weight with increased number of tubers is due to the decrease in each tuber’s share of photosynthetic materials transferred from the leaves.

In terms of biochemical parameters, NaCl treated potato plant exhibited significant proline elevation relative to plants under control conditions (Fig. 3). Enhance proline synthesis in stressed plants was detected in several studies (Fan et al., 2015, Mosaad et al., 2020, Naseem and Bano, 2014, Rabie and Almadini, 2005). The hypothesis that the increase in the proline content under abiotic stress may be refer to the effect of amino acid proline in maintaining the cell water status; thereby, helping the plant to cope and decrease the osmotic potential with the salinity stress (Al-Mansoori et al., 2006, Mousavi et al., 2020).

## 5 Conclusions

In conclusion, we have developed a system for *Solunum tuberosum* regeneration from *in vitro* leaf explants under salt stress. The application of the protocol to *in vitro* regeneration using TDZ was better than BAP of shoot induction. Potato two cultivars differed significantly in their tolerance to salt stress. Victoria cultivar is more tolerant to salt stress than Rosetta cultivar when cultured on MS medium supplemented with 150 mMNaCl. It will facilitate research into the production of stable salt-tolerant plants of potato from *in vitro* selected cells. It may be of economic importance in arid and semi-arid lands of the world and the development of new non-traditional programs for potato breeding. Furthermore, the studied cultivars may be suitable for selection and cultivation in the newly reclaimed land.

## Declaration of Competing Interest

The authors declare that they have no known competing financial interests or personal relationships that could have appeared to influence the work reported in this paper.

## References

[b0005] Abeuova, L.S., Kali, B.R., Rakhimzhanova, A.O., Bekkuzhina, S.S., Manabayeva. S. A., 2020. High frequency direct shoot regeneration from Kazakh commercial potato cultivars. Peer J. *8*:p.e9447.*https://doi.org/10.7717/peerj.9447* 10.7717/peerj.9447 PMC7365135 32742778

[b0010] Ahmad I., Maathuis F.J.M. (2014). Cellular and tissue distribution of potassium: physiological relevance, mechanisms and regulation. J. Plant. Physiol..

[b0020] Al Kharusi L., Assaha D.V., Al-Yahyai R., Yaish M.W. (2017). Screening of date palm (*Phoenix dactylifera* L.) cultivars for salinity tolerance. Forests..

[b0025] Aldhebiani, A, Y., Metwali, E.M., Soliman, H.I., Howladar, S. M., 2018. Response of different date palm cultivars to salinity and osmotic stresses using tissue culture technique. Inter. J. Agric. Biol. 20, 1581-1590.

[b0030] Al-Hussaini Z.A., Yousif S.H.A., Al-Ajeely S.A. (2015). The role of sucrose and light duration on *in vitro* tuberization for two cultivars of potato *Solanum tuberosum* L. Int. J. Curr. Microbiol. Appl. Sci..

[b0035] Ali, S., Khan, N., Nouroz, F., Erum, S., Nasim, W., 2018. Effects of sucrose and growth regulators on the microtuberization of cip potato (*Solanum tuberosum*) germplasm. Pak. J. Bot, 1,763-768.

[b0040] Al-Mansoori T.A., Alaa El-Deen M.N., Caligari P.D. (2006). Evaluation of *in vitro* screening techniques for salt tolerance in date palm. III International Date Palm Conference.

[b0045] Anschütz U., Becker D., Shabala S. (2014). Going beyond nutrition: Regulation of potassium homoeostasis as a common denominator of plant adaptive responses to environment. Plant Physiol..

[b0050] Badoni A., Chauhan J.S. (2009). Microtuber: A Source of germ plasm conservation. Rep. Opin..

[b0055] Coleman W.K., Donnelley D.J., Coleman S.E. (2001). Potato microtubers as research tools: A review. Am. J. Potato Res..

[b0060] Craig D.A., Moller I.S. (2010). Na+ transport in glycophytic plants: what we know and would like to know. Plant Cell Environ..

[b0065] Davenport S.B., Gallego S.M., Benavides M.P., Tomaro M.L. (2003). Behaviour of antioxidant defense system in the adaptive response to salt stress in *Helianthus annuus* L. cells. Plant Growth Regul..

[b0070] Ebadi, M., AIranbakhsh, A., 2011. The induction and growth of potato (*Solanum tuberosum*. L) microtubers (sante cultivar) in response to the different concentrations of 6-benzylaminopurine and sucrose. Afr. J. Biotechnol. 10:10626-10635.

[b0075] El Far M.M. (2007). Optimization of growth conditions during sweet potato micro-propagation. African Potato Association Conference Proceedings.

[b0080] Errabii T., Gandonou C.B., Essalmani H., Abrini J., Idaomar M., Skali-Senhaji N. (2006). Growth, proline and ion accumulation in sugarcane callus cultures under drought-induced osmotic stress and its subsequent relief. Afr. J. Biotechnol..

[b0085] Estrada R., Tovar P., Dodds J.H. (1986). Induction of *in vitro* tubers in a broad range of potato genotypes. Plant Cell Tissue Organ Cul..

[b0090] Etienne H., Berger A., Carron M.P. (1991). Water status of callus from Heveabrasiliensis during induction of somatic embryogenesis. Physiol. Plant..

[b0095] Fan X., Hu H., Huang G., Huang F., Li Y., Palta J. (2015). Soil inoculation with Burkholderia sp. LD-11 has positive effect on water-use efficiency in inbred lines of maize. Plant Soil.

[b0100] Farshadfar E., Jamshidi B., Cheghamirza K., Hashemzadah H. (2012). Evaluation of drought tolerance in bread wheat (*Triticum aestivum* L.) using immature embryo culture. Ann. Biol. Res..

[b0105] Flowers T.J., Munns R., Colmer T.D. (2014). Sodium chloride toxicity and the cellular basis of salt tolerance in halophytes. Ann. Bot..

[b0120] Gambuti A., Rinaldi A., Romano R., Manzo N., Moio L. (2016). Performance of a protein extracted from potatoes for fining of white musts. Food Chem..

[b0125] Gossett D.R., Banks S.W., Millhollon E.P., Lucas M.C. (1996). Antioxidant response to NaCl stress in a control and anNaCl-tolerant cotton cell line grown in the presence of paraquat, buthionine sulfoximine, and exogenous glutathione. Plant Physiol..

[b0130] Gowayed M.H., Al-Zahrani H.S., Metwali E.M. (2017). Improving the salinity tolerance in potato (*Solanum tuberosum*) by exogenous application of silicon dioxide nanoparticles. Int. J. Agric. Biol..

[b0135] Gu R., Liu Q., Pei D., Jiang X. (2004). Understanding saline and osmotic tolerance of *Populus euphratica* suspended cells. Plant Cell Tissue Organ Cul..

[b0140] Hanin M., Ebel C., Ngom M., Laplaze L., Masmoudi K. (2016). New insights on plant salt tolerance mechanisms and their potential use for breeding. Front Plant Sci..

[b0145] Hmidi D., Abdelly C., Ashraf M., Messedi D. (2018). Effect of salinity on osmotic adjustment, proline accumulation and possible role of ornithine-δ-aminotransferase in proline biosynthesis in *Cakile maritima*. Physiol. Mol. Biol. Plants..

[b0150] Horie T., Schroeder J.I. (2004). Sodium transporters in plants, diverse genes and physiological functions. Plant Physiol..

[b0155] Hossain M.J. (1994). *In vitro* propagation of potato (*Solanum tuberosum* L.). J. Plant Tissue. Cult..

[b0160] Idowu P.E., Ibitoye D.O., Ademoyegun O.T. (2009). Tissue culture as a plant production technique for horticultural crops. Afr. J. Biotechnol..

[b0165] Isayenkov, S, V., Maathuis, F. J., 2019. Plant salinity stress: Many unanswered questions remain. Front plant Sci. 10, 80. 10.3389/fpls.2019.00080 PMC6384275 30828339

[b0170] Khalafalla M.M., AbdElaleem K.G., Modawi R.S. (2010). Callus formation and organogenesis of potato (*Solanum tuberosum*L.) cultivar Almera. J. Phytol..

[b0175] Kumlay, A, M., Ercisli, S., 2015. Callus induction, shoot proliferation and root regeneration of potato (*Solanum tuberosum* L.) stem node and leaf explants under long-day conditions. Biotechnol. Biotechnol. Equip. 29, 1075-1084.

[b0180] Liao X., Su Z., Liu G., Zotarelli L., Cui Y., Snodgrass C. (2016). Impact of soil moisture and temperature on potato production using seepage and center pivot irrigation. Agric. Water Manag..

[b0185] Lutts S.M., Almansouri M., Kinet J. (2004). Salinity and water stress have contrasting effects on the relationship between growth and cell viability during and after stress exposure in durum wheat callus. Plant Sci..

[b0190] Martínez-Ballesta M.C., Dominguez-Perles R., Moreno D.A., Muries B., Alcaraz-López C., Bastías E., García-Viguera C., Carvajal M. (2010). Minerals in plant food: effect of agricultural practices and role in human health: A review. Agron. Sustain. Dev..

[b0195] Mashhad S., Moeini M. (2015). The effect of cytokinin and coumarin on *in vitro* micrrotuberization of potato (*Solanum tuberosum* L.) cv. Marfona*. Ludus Vitalis*.

[b0200] Mc Mannus M.T., Bielesk R.L., Caradus J.R., Barker D.J. (2000). Pinitol accumulation in mature leaves of white clover in response to water deficit. Environ. Exp. Bot..

[b0205] Mengs, B., M., Egigu, M., C., Abraha, E., 2018. *In vitro* propagation of sweet potato (*Ipomoea batatas* (L.) Lam.) through lateral bud culture. Int. J. Innovative Pharm. Sci. Res. 6, 1-12.

[b0210] Metwali E.M.R., Kadasa N.M.S., Soliman H.I.A., Almaghrabi O.A., Fuller M.P. (2020). *In vitro* propagation of date palm cultivars Magdoul and Safwai through somatic embryogenesis. Int. J. Agric. Biol..

[b0215] Metwali E.M., Soliman H.I., Fuller M.P., Al-Zahrani H.S., Howladar S.M. (2015). Molecular cloning and expression of a vacuolar Na+/H+ antiporter gene (AgNHX1) in fig (Ficus carica L.) under salt stress. Plant Cell Tissue Organ Cult..

[b0220] Miki Y., Hashiba M., Hisajima S. (2001). Establishment of salt stress tolerant rice plants through step up NaCl treatment *in vitro*. Biol. Plant.

[b0225] Mitra S. (2012). Nutritional status of orange-fleshed sweet potatoes in alleviating vitamin A malnutrition through a food-based approach. J. Nutr. Food Sci..

[b0230] Mohamed M.F., Abdalla M.M.A., Damarany A.A.M. (2007). Differential axillary-bud proliferation responses of two sweet potato cultivars to benzyl adenine and thidiazuron. Ass. Univ. Bull. Environ. Res..

[b0235] Mohapatra P.P. (2018). *In vitro* multiplication and microtuberization of *Slanumtuberosum* using dif-ferent growth regulators. Vegetos.

[b0240] Mosaad I.S., Serag A.H., Moustafa-Farag M., Seadh A.K. (2020). Effect of exogenous proline application on maize yield and the optimum rate of mineral nitrogen under salinity stress. J. Plant Nutr..

[b0245] Motallebi-Azar A., Kazemiani S., Yarmohamadi F. (2013). Effect of sugar/osmotic levels on *in vitro*microtuberization of potato (*Solanum tuberosum* L.). Russ. Agric. Sci..

[b0250] Mousavi S.N., Ebadi M., Khorshidi M., Hokmabadi H., Masoudian N. (2020). Effect of salinity on photosynthetic and enzymatic activities and tuberization yield in the genotype of potato cultivar Agria under *in vitro* conditions. J. Neotropical Agric*.*.

[b0255] Munns R. (2002). Comparative physiology of salt and water stress. Plant Cell Environ..

[b0260] Murashige T., Skoog F. (1962). A revised medium for rapid growth and bioassays with tobacco tissue cultures. Physiol. Plant..

[b0265] Mutasim M.K., Khadiga G.A.E., Rasheid S.M. (2010). Callus formation and organogenesis of potato (*Solanumtuberosum* L.) cultivar Almera. J. Phytol..

[b0270] Naseem H., Bano A. (2014). Role of plant growth-promoting rhizobacteria and their exopolysaccharide in drought tolerance of maize. J. Plant Interact..

[b0275] Nistor A., Campeanu G., Atanasiu N., Chiru N., Karaconyi D. (2010). Influence of genotype on microtuber production. Not. Bot. Horti. Agrobot. Cluj..

[b0280] Olmos E., Hellín E. (1996). Mechanisms of salt tolerance in a cell line of *Pisum sativum*: Biochemical and physiological aspects. Plant Sci..

[b0285] Pasternak, T., Prinsen, E., Ayaydin, F., Miskolczi, P., Potters, G., Asard, H., Van Onckelen, H., Dudits, AFehér, D., 2002. The role of auxin, pH and stress in the activation of embryogenic cell division in leaf protoplast-derived cells of alfalfa (*Medicago sativa* L.). Plant Physiol. 129, 1807-1819. 10.1104/pp.000810 PMC166769 12177494

[b0290] Pérez-Tornero, O., Egea, AVanoostende, J., Burgos, L., 2000. Assessment of factors affecting adventitious shoot regeneration from *in vitro* cultured leaves of apricot. Plant Sci. 158, 61-70. 10.1016/s0168-9452(00)00303-4 10996245

[b0295] Pour M.S., Omidi M., Majidi I., Davoodi D., Tehrani P. (2010). *In-vitro* plantlet propagation and microtuberization of meristem culture in some wild and commercial potato cultivars as affected by NaCl. Afr. J. Agric. Res..

[b0300] Rabie G., Almadini A. (2005). Role of bioinoculants in development of salttolerance of *Viciafaba* plants under salinity stress. Afr. J. Biotechnol..

[b0305] Rahman M., Islam R., Hossain M., Haider S. (2008). Differential response of potato under sodium chloride stress conditions *in vitro*. J. Biosci..

[b0310] Ranalli P. (2007). The Canon of Potato Science: 24. Microtubers. Potato Res..

[b0315] Ranalli P., Bassi F., Ruaro G., Del Re P., Dicandilo M., Mandolino G. (1994). Microtuber and minituber production and field performance compared with normal tubers. Potato Res..

[b0320] Rathore M.S., Patel P.R., Siddiqui S.A. (2020). Callus culture and plantlet regeneration in date palm (*Phoneix dactylifera* L.): An important horticultural cash crop for arid and semi-arid horticulture. Physiol. Mol. Biol. Plants..

[b0325] Reetika S., Sabina A., Nishi K. (2019). Genetic homogeneity appraisal of *in vitro* plants regenerated from leaf explants of *Sapindus mukorossi* using RAPD and ISSR molecular markers. Res. J. Biotechnol..

[b0330] Roodbar-Shojaei T., Omidi M., Sepahvand N.A., Mohammadi A., Fazeli A., Motallebi-Chaleshtori R., Abbasi-Sahebi A. (2010). Production of virus free commercial potato mini-tuber by meristem culture. Biharean Biol..

[b0335] Sajid Z., Aftab F. (2014). Plant regeneration from in vitro-selected salt tolerant callus cultures of *Solanum tuberosum* L. Pak. J. Bot..

[b0340] Sané, D., Aberlenc-Bertossi, F., Diatta, L.I.D., Guèye, B., Daher, A., Sagna, M., Duval, Y., Borgel, A., 2012. Influence of growth regulators on callogenesis and somatic embryo development in date palm (*Phoenix dactylifera* L.) Sahelian cultivars. Sci. World J. 2012; Article 837395. 10.1100/2012/837395 PMC3353711 22629211

[b0345] Schween G., Schwenkel H.G. (2003). Effect of genotype on callus induction, shoot regeneration, and phenotypic stability of regenerated plants in the greenhouse of Primula ssp. Plant Cell Tissue Organ cult..

[b0350] Shankhdhar D., Shankhdhar S.C., Mani S.C., Pant R.C. (2000). *In vitro* selection for salt tolerance in rice. Biol. Plant..

[b0355] Silva J.A.B., Otoni W.C., Martinez C.A., Dias L.M., Silva M.A.P. (2001). Microtuberization of Andean potato species (Solanum spp.) as affected by salinity. Sci. Hortic..

[b0360] Skoog, D.A., West, Holler, D.M., Crouch, S.R., 2007. Analytical Chemistry: An Introduction, New Age International PVT, UK 594-631 pp.

[b0365] Sobieh S., Rashad T., Adam Z., Elfiki A., Awad A. (2019). Salt stress induces changes in genetic composition, proline content and subcellular organization in potato (*Solanum tuberosum* L.). Egypt. J. Bot..

[b0370] Steel G.D., Torrie J.H., Dickey D.A. (1997).

[b0375] Szabados L., Savoure A. (2010). Proline: A multifunctional amino acid. Trends Plant Sci.

[b0380] Thirukkumaran G., Ntui V.O., Khan R.S., Mii M. (2009). Thidiazuron: an efficient plant growth regulator for enhancing Agrobacterium-mediated transformation in Petunia hybrida. Plant Cell Tissue Organ cult..

[b0385] Twaij B.M., Jazar Z.H., Hasan M.N. (2020). Trends in the use of tissue culture, applications and future aspects. Int. J. Plant Biol..

[b0390] Uranbey S. (2005). Comparison of kinetin and 6-benzyladene (BA) on *in vitro* microtuberization of potato under short days conditions. J. Agric. Sci..

[b0395] Visser R., Vreugdenhil D., Hendriks T., Jacobsen E. (1994). Gene expression and carbohydrate content during stolon to tuber transition in potatoes (*Solanum tubrosum*). Physiol. Plant..

[b0400] Wikandari R., Manikharda B.S., Ningrum A., Taherzadeh M.J. (2021). Application of cell culture technology and genetic engineering for production of future foods and crop improvement to strengthen food security. Bioengineered.

[b0405] Wu H., Zhang X., Giraldo J.P., Shabala S. (2018). It is not all about sodium: revealing tissue specificity and signaling roles of potassium in plant responses to salt stress. Plant Soil..

[b0410] Yagiz A.K., Yavuz C., Tarim C., Demirel U., Caliskan M.E. (2020). Effects of growth regulators, media and explant Ttypes on Microtuberization of potato. Am. J. Potato. Res..

[b0415] Zhang Z., Mao B., Li H., Zhou W., Takeuchi Y., Yoneyama K. (2005). Effect of salinity on physiological characteristics, yield and quality of microtubers *in vitro* in potato. Acta Physiol. Plant.

[b0420] Zuo J.R., Niu Q.W., Frugis G., Chua N.H. (2002). The WUSCHEL gene promotes vegetative-to-embryonic transition in Arabidopsis. Plant J..

